# Managing the incidence of selective reporting bias: a survey of Cochrane review groups

**DOI:** 10.1186/s13643-015-0070-y

**Published:** 2015-06-13

**Authors:** Emma K Reid, Aaron M Tejani, Lawrence N Huan, Gregory Egan, Cait O’Sullivan, Alain D Mayhew, Monisha Kabir

**Affiliations:** Pharmaceutical Sciences, Vancouver General Hospital, 855 West 12th Avenue, Vancouver, BC V5Z 1M9, Canada; Therapeutics Initiative (Faculty of Medicine), University of British Columbia, 2176 Health Sciences Mall, Vancouver, BC V6T 1Z3, Canada; Department of Pharmacy, Richmond General Hospital, 7000 Westminster Highway, Richmond, BC V6X 1A2, Canada; Island Health Clinical Pharmacy Programs, 375 Second Avenue, Campbell River, BC V9W 3V1, Canada; Knowledge Synthesis Group, Cochrane Methods—Bias, Ottawa Hospital Research Institute, 501 Smyth Road, Ottawa, ON K1H 8L6, Canada; Department of Biology, University of Ottawa, 75 Laurier Ave E, Ottawa, ON K1N 6N5, Canada

**Keywords:** Selective reporting bias, Outcome reporting bias, Systematic review, Cochrane Collaboration

## Abstract

**Background:**

Selective reporting bias (SRB), the incomplete publication of outcomes measured or of analyses performed in a study, may lead to the over- or underestimation of treatment effects or harms. Cochrane systematic reviews of interventions are required to assess the risk of SRB, achieved in part by applying the Cochrane risk of bias tool to each included randomised trial. The Cochrane Handbook outlines strategies for a comprehensive risk of bias assessment, but the extent to which these are followed by Cochrane review groups (CRGs) has not been assessed to date. The objective of this study was to determine the methods which CRGs require of their authors to address SRB within systematic reviews, and how SRB risk assessments are verified.

**Methods:**

A cross-sectional survey was developed and distributed electronically to the 52 CRGs involved in intervention reviews.

**Results:**

Responses from 42 CRGs show that the majority refer their authors to the Cochrane Handbook for specific instruction regarding assessments of SRB. The handbook strategies remain variably enforced, with 57 % (24/42) of CRGs not requiring review authors to search for included trial protocols and 31 % (13/42) not requiring that contact with individual study authors be attempted. Only half (48 %, 20/42) of the groups consistently verify review authors’ assessments of the risk of SRB to ensure completeness.

**Conclusions:**

A range of practices are used by CRGs for addressing SRB, with many steps outlined in the Cochrane Handbook being encouraged but not required. The majority of CRGs do not consider their review authors to be sufficiently competent to assess for SRB, yet risk of bias assessments are not always verified by editors before publication. The implications of SRB may not be fully appreciated by all CRGs, and resolving the identified issues may require an approach targeting several steps in the systematic review process.

**Electronic supplementary material:**

The online version of this article (doi:10.1186/s13643-015-0070-y) contains supplementary material, which is available to authorized users.

## Background

Selective reporting bias (SRB) is a form of reporting bias in which certain components of conducted research are not fully presented based on the nature or direction of the results [1]. SRB may include the incomplete publication of a study analysis (e.g. subgroup), inconsistencies in predefined measurement scales or time-points for data collection, or the re-ranking of outcomes previously defined as primary or secondary [1–5]. A major component of SRB is outcome reporting bias (ORB), which occurs when only a subset of outcomes originally measured in a study is selected for publication [2, 3].

An analysis of randomised controlled trials (RCTs) demonstrated that 40–62 % of trials had changed, omitted or introduced one or more primary outcomes for publication, indicating a high prevalence of SRB [3]. There is also evidence to suggest that study outcomes which are statistically significant are more likely to be published, with estimated odds for publication being two to four times greater than those not reaching significance [6]. The distorted presentation of study outcomes gives rise to a potential over- or underestimation of treatment effects or harms [2–4, 7].

The impact of ORB in RCTs is inherently carried forward when results are meta-analysed and included in systematic reviews [8]. In the ORBIT study, Kirkham et al. analysed the prevalence of ORB within a cohort of Cochrane systematic reviews and reported that 34 % of Cochrane reviews included one or more trials with a high suspicion for ORB in its analysis [2]. In an exploratory sensitivity analysis of Cochrane reviews with a single meta-analysis of the primary outcome, close to one fifth of those which reported statistically significant results became non-significant once adjusted for the presence of ORB in its primary studies [2]. In addition, almost one quarter (23 %) of the results in this subgroup with statistical significance would have exaggerated the treatment effect by 20 % or more [2].

The Cochrane Collaboration is an international network of researchers, health practitioners and patient advocates, aiming to produce and publish credible and accessible health information free from conflicts of interest. Cochrane reviews are recognised globally as being systematic reviews of the highest standard in evidence-based health care [9]. For Cochrane systematic reviews, the Methodological Expectations of Cochrane Intervention Reviews (MECIR) standards have been developed to ensure high-quality and consistent review development and reporting [10]. All Cochrane reviews must include an assessment of risk of bias [10]. Cochrane review authors are strongly encouraged to approach this assessment in a domain-based fashion by applying the Cochrane risk of bias tool, which addresses several domains of bias (i.e. sequence generation, allocation concealment, blinding of participants and personnel, blinding of outcome assessment, incomplete outcome data, selective reporting) for each trial considered for inclusion [4]. Instructions for performing the assessment of risk of bias are outlined in the Cochrane Handbook for Systematic Reviews of Interventions, including factors to consider when determining if a domain should be judged as “low,” “high” or “unclear” risk of bias [4]. Briefly, review authors are asked to make the judgement and include a description of how it was made, referencing a direct quote or describing the text from which the judgement was concluded, and include a comment supporting the judgement. For the SRB domain, the handbook provides instruction for the assessment of selective outcome reporting. It encourages review authors to construct a table or “matrix” of trials with their reported outcomes to better identify gaps in reported outcomes. Review authors are also encouraged to compare a trial’s protocol or registry data to its final publication to assess for discrepancies in outcome reporting and to contact trial authors for unreported outcome data whenever possible [4].

It is expected that review authors incorporate their risk of bias assessment into their final analysis. Judgement about which bias domains have the greatest potential to affect their particular review’s results is a critical consideration for the review author [4]. Strategies to address the implications of the risk of bias include presenting multiple analyses of data accounting for different types of bias (e.g. worst-case scenario analysis for missing patient data), restricting the primary analysis to those studies only carrying a low (or unclear) risk of bias (e.g. sensitivity analysis for blinded vs open label studies), or presenting all studies accompanied by a detailed narrative discussion of the risk of bias [4].

The consideration of the review authors, editors and staff from Cochrane review groups (CRGs) regarding this assessment of risk of bias is crucial for the publication of reliable, balanced and objective systematic reviews. Cochrane review authors, however, may not be reliably detecting ORB within trials [2]. Under-recognition of ORB has recently been demonstrated in a study evaluating 30 well-respected biomedical journals [11]. In this survey-based analysis, half of responding journals indicated that ORB was uncommonly or never detected in their journal’s editorial procedures and 64 % of the journals had no method at all in place for detecting ORB [11].

Gaining knowledge of the understanding of and appreciation for SRB by Cochrane review groups and the methods they implement to account for the effect of SRB from primary studies in systematic reviews will help inform how current methods may be improved.

The primary aim of this research was to identify the practices of CRGs for minimising SRB in their systematic reviews. This includes identifying the instruction provided by CRGs to their review authors to assess for and address selective outcome reporting in the various stages of a systematic review (its protocol, the final review, and subsequent updates). In addition, the specific methods of each of the CRGs for verifying the completeness of the assessment of SRB would be determined. A secondary component of the research was to explore how fully CRGs believe their review authors understand and appreciate SRB.

## Methods

A cross-sectional survey with 21 questions (see Additional file 1) was developed with questions pertaining to the following seven themes:

1. Instruction provided to authors regarding SRB
2. Consideration for SRB in systematic review protocol
3. Assessment of risk of SRB within the RCTs in a systematic review
4. Assessment of risk of SRB on the level of the systematic review
5. Assessment of risk of SRB in updates of systematic reviews
6. Importance of SRB to review authors
7. General

The majority (11/21) of questions addressed the third theme (assessment of risk of SRB within the RCTs in a systematic review). The responses to more than half of the questions were categorical, with the number of categories ranging from three to five. The option for free-text (“other” or “sometimes”) responses, however, was also available in almost all questions. The survey content was piloted for relevance with two active members of the Cochrane Bias Methods Group with knowledge of systematic reviews and SRB. Thereafter, three scientists with no Cochrane involvement piloted the survey with regards to readability. The questions were further adjusted based on these individuals’ feedback, to ensure relevance and clarity.

The survey was distributed to the 52 Cochrane review groups who conduct reviews of clinical interventions excluding only the Methodology Review Group. The managing and coordinating editor(s) in each group were invited via email to participate in the survey. These editors could choose to respond themselves or delegate the response of the survey to another group member. The intended respondent was an individual regularly involved in reviewing assessments of risk of bias in systematic reviews or having sufficient knowledge of this process to accurately represent the practices of the CRG as a whole. Consultation between group members was encouraged so that the responses would capture group practices rather than individual variations. One survey per CRG was accepted.

Distribution of the survey began in December 2013, and collection of survey submissions continued until March 2014. Up to three reminder emails were sent at approximately two-week intervals.

Data were collected and tabulated with no identifying information via the FluidSurveys™ survey platform. Descriptive statistics were used to analyse survey responses. Five authors independently assessed all survey submissions, and a meeting was held to discuss the responses, including open-ended questions, to identify important issues and patterns. The final decision on issues and patterns to discuss further was determined by consensus.

Ethical approval for the study was received from both the Behavioural Research Ethics Board at the University of British Columbia and the Fraser Health Services Authority, Vancouver, Canada.

## Results

Of the 52 CRGs invited to participate in the study, 42 provided responses (81 %). In the one instance where two completed surveys were received from one group, the submitting group members were contacted and asked to clarify via a single survey submission. Open-ended responses accounted for approximately one fifth of all responses. The meeting of five authors to discuss survey responses identified trends in the open responses. Any key issues or patterns that were identified in categorical and open survey questions will be discussed in the categories below. Full results, including all open responses, are available via Figshare (http://dx.doi.org/10.6084/m9.figshare.1421975).

### Instruction provided to authors regarding SRB

The majority of CRGs refer their review authors to the Cochrane Handbook for instruction regarding determining outcomes for analysis (86 % [36/42]), selecting trials for inclusion (78 % [33/42]) and conducting their assessment of SRB (86 % [36/42]). There was also a trend for referring review authors to group-specific instruction or guidelines.

### Consideration for SRB in systematic review protocol

Almost all CRGs require a statement in their systematic review protocols that the Cochrane risk of bias tool will be applied to all included RCTs (88 % [37/42]). Only 38 % (16/42) require a description within the protocol of how the assessment of the risk of SRB will be incorporated into the results and/or discussion of the review.

### Assessment of risk of SRB within the RCTs in a systematic review

Three quarters of CRGs stated that review authors are responsible for performing assessments of risk of SRB (76 % [32/42]). Editorial staff members also play a role in performing SRB assessments, with managing editor involvement reported in 50 % [21/42] of CRGs. Some open responses indicated methodological and statistical editors perform assessments of SRB.

Less than half of CRGs report they always review risk of bias assessments in submitted systematic reviews (48 % [20/42]). Nineteen percent (8/42) of CRGs report never verifying risk of bias assessments for SRB specifically or verifying only that the risk of bias tool is completed. It appeared from open responses that risk of bias assessments were more likely to be verified if the risk of bias tool appeared to be filled out incorrectly by review authors. CRGs reported a very wide range (from 0–100 %) for the number of reviews with SRB assessments that require revision before publication. The most frequently reported requirement for revision was the risk of bias tool being incomplete or discrepant.

Fifty-seven percent (24/42) of CRGs do not require review authors to seek out trial protocols as a step in performing their risk of SRB assessment. The time required to perform the search and lack of availability of protocols were indicated as barriers to performing this task. Additionally, 31 % (13/42) of CRGs do not require review authors to attempt to contact trial authors regarding the completeness of outcome data. In some cases, this step was reported to be encouraged, but not required. The creation of a “matrix” of reported trial outcomes amongst trials included in the systematic review was only reported by 14 % of CRGs (6/42), though a few additional groups indicated that data extraction forms or other group-specific tables may be used for this purpose.

The incorporation of an assessment of the risk of SRB into the results or discussion section of the systematic review is required by 45 % (19/42) of CRGs. If CRGs reported that the incorporation is “sometimes” required, it was typically only if SRB had been identified within the review but not when SRB assessments were done and did not find evidence of SRB.

### Assessment of risk of SRB on the level of the systematic review

Fifty-two percent (22/42) of CRGs report consistently assessing for the selective inclusion of RCTs in a final systematic review (i.e. is the subset of data included fully representative of available trial data?). The systematic review protocol is compared to the final review for consistency in defined outcome analyses by 86 % (36/42) of CRGs. Eighty-six percent (36/42) of CRGs reported that if a discrepancy in a protocol-defined outcome is recognised, a justification for the change is included in the methods (or more specifically, the “Differences between protocol and review”) section of the systematic review.

### Assessment of risk of SRB in updates of systematic reviews

Despite being considered a mandatory MECIR standard, only three quarters (31/42) of CRGs reported insisting that the Cochrane risk of bias tool be applied to *all* RCTs included when performing an update of a systematic review. In several instances, it was reported that only newly incorporated RCTs were subject to assessment with the risk of bias tool.

### Importance of SRB to review authors

**Fig. 1:**
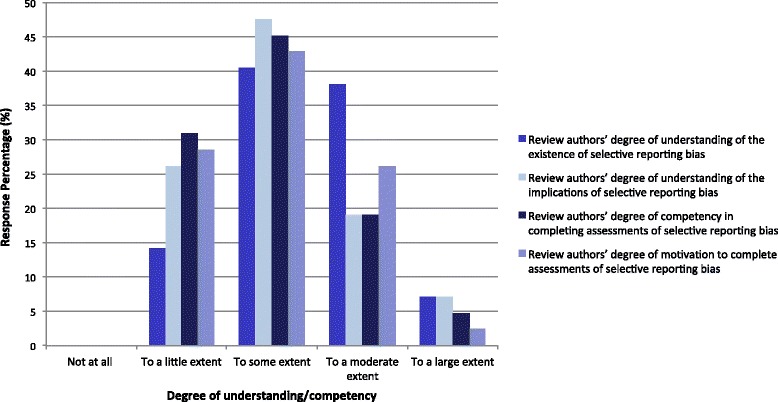
Q20: How do you rate your review authors’ understanding of SRB?

CRGs were asked to classify their authors’ degree of understanding of the existence of SRB, the implications of SRB, their competency in completing and motivation for completing assessments of SRB (see Fig. 1). Authors were perceived as having a greater degree of understanding of the *existence* of SRB than the *implications* of SRB (45 vs 26 % had a moderate or large extent of understanding, respectively). Thirty-one percent of groups reported their authors had a “little” degree of competency in performing SRB assessments. Only one CRG rated their review authors as having a “large” degree of motivation to complete SRB assessments.

When considering only those CRGs who reported that they refer their review authors to the Cochrane Handbook for instruction for assessing SRB, only 23 % (9/39) classified their review authors to be moderately to largely competent in performing SRB assessments.

### General

Four of 42 CRGs reported having frequently contacted the Cochrane Bias Methods Group for guidance in the previous year. Over 60 % of CRGs report never having contacted the Cochrane Bias Methods Group in this time.

## Discussion

Our survey in full provides an abundance of information. Many of the results identify issues regarding the extent to which SRB is recognised and appreciated by CRGs. The focus of this discussion is on the issues for which we could identify a possible course of action for improvement.

The completeness of SRB assessments in systematic reviews relies on a comprehensive evaluation of the presence of bias in included RCTs. Despite the Cochrane Handbook being the primary resource for CRGs for instructing risk of bias assessments, its recommendations for evaluating SRB are implemented to a variable extent by different groups.

An essential step in assessing SRB is searching for each trial’s protocol and comparing the originally defined outcomes with those in the final publication for completeness. It is reasonable to hypothesise that, with 57 % of CRGs not requiring that trial protocols be identified and reviewed, this disparity contributes to frequent “unclear” risk of bias assessments in the SRB domain. As time and resources involved in protocol and trial registry searches are identified barriers to completing the step, it would be advantageous to increase the guidance in the Cochrane Handbook regarding streamlining these searches. A greater availability of trial protocols in the public domain would also help alleviate this strain [12]. Fortunately, the use of trial registries has increased since the introduction of the first trial registry platform (i.e. ClinicalTrials.gov) in 2000, and the implementation of policies encouraging the registration of trial design information prior to patient enrolment by the International Committee of Medicine Journal Editors (ICMJE) and the United States Food and Drug Administration (US FDA) in 2005 [13] and 2007 respectively [14]. Given the important implications of SRB and the existing resources to access many protocols, we feel the protocol search is a step that should be mandatory in all systematic reviews, despite its additional time requirement.

The Cochrane Handbook recommends that authors of trials be contacted in attempt to access all pre-specified outcome data in the case of discrepancies [4], yet our survey suggests almost one third of CRGs make no attempt to do so. We also consider this step critical to making an informed judgement regarding the bias risk for SRB.

After an assessment of the risk of SRB has been made, it is prudent to incorporate these findings into the results and discussion of the systematic review in the manner defined in the review protocol. The incorporation of these assessments was reported to be a mandatory step before review publication by 45 % of CRGs. Some groups indicated that a comment on SRB would be made only if the bias was recognised to be present. Considering the potentially extensive implications of SRB on the findings of any trial, we feel striving to provide as much detail as possible around its presence *or* absence should be a priority in all systematic reviews to maximally inform readers.

A lack of understanding and appreciation for the implications of SRB, as well as how one may comprehensively assess for it, may very well be the root of the aforementioned issues. In a survey administered by Savovic and colleagues, SRB was identified by Cochrane review authors as being one of the most difficult types of bias to analyse in risk of bias assessments [15]. Further, our survey included a set of questions inquiring, in general, how well-equipped each CRG believes their review authors are in addressing SRB in their reviews. Only 24 % of groups consider their authors to be moderately or largely capable of performing SRB assessments, which suggests a perceived knowledge gap for the review authors. Despite this, only 48 % of CRGs reported that assessments of risk of SRB in their reviews are consistently (“always”) verified before publication, suggesting this is a discrepancy worth addressing.

We feel the approach to addressing the SRB-related issues recognised in our survey would require targeting several steps in the systematic review process. We fully acknowledge that our survey did not address the training and skills of review authors directly and focused instead on editorial staff. It remains prudent, however, to include review authors in our proposed approach to minimise the impact of SRB in systematic reviews.

We suggest starting from the beginning, during the training of Cochrane review authors. We propose that standard author training be structured in a way that includes a focused review of SRB, including a focus on its implications and the precise steps for its proper assessment. Education could also go beyond this initial training, with the introduction of online learning modules or refresher courses for assessing risk of bias for both new and more seasoned review authors.

For the assessment of risk of SRB itself, the steps outlined in the Cochrane Handbook should more consistently be followed. Optimal mandatory steps would require editorial staff to enforce the protocol or trial registry search and the contact attempts with original trial authors. By doing this, trial exclusion due to the lack of relevant outcome data as a result of SRB could be minimised.

**Fig. 2:**
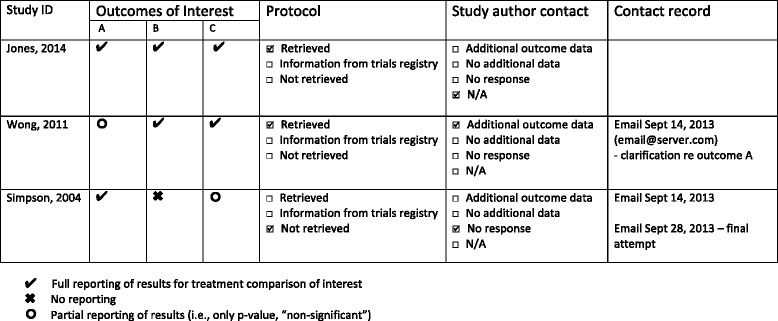
Proposed matrix-based data extraction form. (Adapted from Dwan et al. [16])

The handbook-described organisation of trials and their reported outcomes in a matrix was identified to be an underused strategy. One suggestion that may help review authors take a systematic approach to assessing SRB is by each CRG implementing a standardised matrix-based data extraction form (Fig. 2). On this form, review authors would not only record the trial outcomes as described previously [4, 16] but would also document both the protocol search and author contact attempts. This would help ensure each of these steps is performed and would make the subsequent justification for the risk of bias assessment very transparent. The proposed matrix form outlines actions that need to take place in a checklist format. This format could increase clarity for review authors and facilitate the editorial process for CRGs by presenting the data in an intuitive and concise manner. This would allow CRG editorial staff to quickly assess and verify the work of the review authors in assessing SRB.

**Fig. 3:**
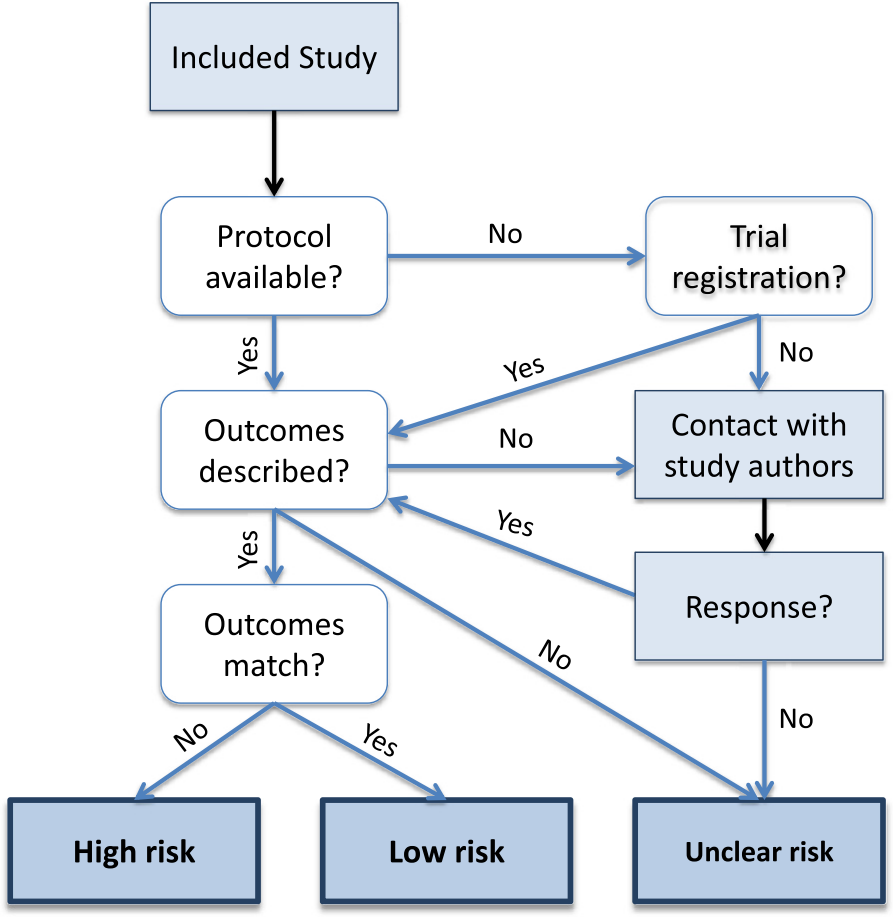
Proposed algorithm for judging risk of selective reporting bias in primary studies

To help clarify the approach of review authors for making final risk of bias judgements for SRB, we suggest an algorithm that lays out each step in the process (Fig. 3). If the protocol of an included study is not available, for example, the ensuing step is to check trial registries to locate outcome data. If no such data are found, an attempt to contact study authors is made. If the protocol or initial outcome data are provided, a comparison to final reported outcomes is made and the consistency determines “high” or “low” risk of SRB. If no response is received from the study authors, an “unclear” judgement is selected.

In order to verify the quality of risk of bias assessments for SRB in a given systematic review, a specified member of editorial staff could be delegated to double-check the primary work. This could be performed as a random sample of a certain percentage, e.g. 10 %, of included trials, as a means for quality assurance.

### Strengths and limitations

The high survey response rate (81 %) is an important strength of our study. Respondents were intended to be CRG staff members or authors with personal involvement or familiarity of risk of bias assessment practices within their group, with discussion amongst members encouraged. Collecting only a single survey submission from each CRG, however, may limit the scope of the information provided to an individual viewpoint rather than reflect the practices of all authors. Additionally, the skill level of review authors themselves for addressing SRB was not directly assessed and instead was reported only as how it was perceived by the survey respondent. This would be better investigated in a secondary study questioning review authors and sampling their reviews directly.

The use of mainly categorical responses for our survey questions may limit the interpretation of the full scope of each response, but the option to elaborate with a free-form response was used to minimise this. With any survey, there is the possibility of misinterpretation of the questions; piloting of our survey questions was performed to lessen this potential. The scope of our survey is limited to a single component of the Cochrane risk of bias tool, which only represents a small portion of the labours of writing and updating Cochrane reviews [15]. Future research is aimed at determining practices of Cochrane review authors themselves for assessing SRB within a sample of reviews, and also exploring other bias domains.

### Application

This study will be of relevance to Cochrane review and methodology groups as well as non-Cochrane systematic reviewers. It will also provide insight for future updates to the Cochrane risk of bias tool and the Cochrane Handbook. It is of importance to the Strengthening the Reporting of Observational studies in Epidemiology (STROBE) efforts [17], the use of the Prospective Registering of Systematic Reviews (PROSPERO) [18], the development of core outcome sets for the Core Outcome Measures in Effectiveness Trials (COMET) Initiative [19], the use of the Consolidated Standards of Reporting Trials (CONSORT) Statement [20], and the Preferred Reporting Items for Systematic Reviews and Meta-Analyses (PRISMA) Statement [21].

## Conclusions

Our study indicates that the recommendations in the Cochrane Handbook for assessing SRB are variably enforced by CRGs. The majority of CRGs do not consider their review authors sufficiently competent to assess for SRB, yet risk of bias assessments are not consistently verified by editors before publication. The implications of SRB may not be fully appreciated by all CRGs, and resolving the identified issues may require a multi-faceted approach targeting several steps in the systematic review process.

## Additional file

Additional file 1:
**Survey Questions.** Copy of original survey instructions and questions for reference.

## Abbreviations

SRB: selective reporting bias
ORB: outcome reporting bias
RCTs: randomised controlled trials
MECIR: Methodological Expectations of Cochrane Intervention Reviews
CRG: Cochrane review group
ICMJE: International Committee of Medicine Journal Editors
US FDA: United States Food and Drug Administration
STROBE: Strengthening the Reporting of Observational studies in Epidemiology
PROSPERO: Prospective Registering of Systematic Reviews
COMET: Core Outcome Measures in Effectiveness Trials
PRISMA: Preferred Reporting Items for Systematic Reviews and Meta-Analyses
