# Supplementary material for: Managing the incidence of selective reporting bias: a survey of Cochrane review groups
Source: Syst Rev. 2015 Jun 13;4:85. doi: 10.1186/s13643-015-0070-y (PMC4472158; doi:10.1186/s13643-015-0070-y)
Supplement: Additional file 1: — Survey Questions. Copy of original survey instructions and questions for reference. [file 13643_2015_70_MOESM1_ESM.pdf]

## Survey Questions

### Instructions for Completing Survey

Please read each survey question carefully and select the response(s) that best represent(s) the practices of your review group. For some questions, multiple responses may be selected. A response is selected by clicking the preceding box. For questions requesting descriptive text or elaboration upon a selected answer, please use the box provided to type a description with as much detail as possible.

If you prefer to complete this survey in a language other than English, please contact investigator Emma Reid at emma.reid@vch.ca and an effort will be made to accommodate the request.

The following definitions should be considered when interpreting survey questions. Please note that these definitions will be repeated throughout the survey, at the bottom of each page. When terms within questions appear underlined please refer to these definitions.

Selective reporting bias (SRB): The publication of a subset of the originally recorded outcome variables in a study. Also known as "outcome reporting bias."

Review authors: The authors of Cochrane reviews.

Trial authors: The authors of RCTs or studies considered for inclusion in Cochrane reviews.

Editorial team: The members of the Cochrane review group responsible for the revision and editing of submitted protocols, systematic reviews and updates of systematic reviews for publication.

Editor: The group member responsible for assessing the specified aspect of a systematic review. Examples include a Contact Editor, Managing Editor or Coordinating Editor.

Assessment of risk of bias ("risk of bias assessment"): The steps taken to assess the likelihood that studies included in the systematic review contain sources of bias. May include, but is not limited to, the completion of the Cochrane risk of bias tool.

Assessment of selective reporting bias: A component of the assessment of risk of bias; the steps taken to specifically assess the likelihood of selective reporting bias within a systematic review.

Key outcome: An outcome identified as an important measure of efficacy or safety for a particular intervention, expected to be included in the study of that intervention.

**Instructing Review Authors to Minimize Selective Reporting Bias**

**1. What instruction does your Cochrane review group provide to review authors regarding determining outcomes for analysis within their systematic review? Select all that apply.**

□ No specific instruction provided

□ Referred to instruction in Cochrane Handbook

□ Specifically instructed to define primary and secondary outcomes prior to study commencement

□ Specifically instructed that key outcomes typically measured in studies of the topic of interest be included as outcomes for analyses

□ Specifically instructed to define acceptable outcome measures (scales, diagnostic criteria) prior to study commencement

□ Specifically instructed to determine the time---frame or time points for the measurement of outcomes prior to study commencement

□ Specifically instructed that at least two individuals independently extract outcome data from each trial

□ Other. Please elaborate: ____________________

**2. What instruction does your Cochrane review group provide to review authors regarding selecting trials to include in their systematic review in a manner that minimizes the risk of selective reporting bias? Select all that apply.**

□ No specific instruction provided

□ Referred to instruction in Cochrane Handbook

□ Specifically instructed that at least two individuals independently perform the study selection process

□ Specifically instructed to note the presence or absence of key outcomes within trials identified in the search

□ Specifically instructed to contact trial authors to identify unpublished outcomes when considering excluding a trial from the systematic review

□ Other. Please elaborate: ____________________

**3. What instruction does your Cochrane review group provide review authors regarding how to conduct their assessment of selective reporting bias for randomized controlled trials (RCTs)? Select all that apply.**

□ No specific instruction provided

□ Referred to instruction in Cochrane Handbook

□ Referred to instruction provided on group---specific resource (i.e., website)

□ Specifically instructed that review authors apply the Cochrane risk of bias tool to each included RCT

□ Specifically instructed that at least two individuals independently apply the Cochrane risk of bias tool to each included RCT

□ Specifically instructed to search for trial protocols to compare with final publications they are considering for inclusion in systematic review

□ Specifically instructed to contact trial authors for any necessary information to complete the Cochrane risk of bias tool

□ Other. Please elaborate: ____________________

**Considerations for SRB within the Protocol of a Systematic Review**

**4. What is the minimum amount of information your editorial team requires review authors to provide in a protocol regarding the assessment of selective reporting bias? Select all that apply.**

□ No comment on assessment of selective reporting bias

□ Statement that the Cochrane risk of bias tool will be applied to each included RCT

□ Description of measures to be taken to adjust for selective reporting bias beyond the application of the Cochrane risk of bias tool

□ Statement that the assessment of selective reporting bias will be interpreted within the Results and/or Discussion of the systematic review

□ Statement of how the assessment of selective reporting bias will be incorporated into the study analyses (i.e., stratification of analyses based on risk of bias, inclusion of trials only at low risk of bias)

□ Other specifications. Please elaborate: ____________________

**Assessment of Risk of SRB of RCTs within the Systematic Review**

**5. Who is responsible for reviewing assessments of risk of bias within a systematic review? Select all that apply.**

□ No particular individual designated

□ Authors of systematic review

□ All peer reviewers of systematic review

□ Specifically designated peer reviewers of systematic review

□ Contact Editor

□ Managing Editor

□ Coordinating Editor

□ Other. Please elaborate: ____________________

**6. How often are the assessments of selective reporting bias reviewed to ensure completeness (by the individual[s] indicated in question 5)? Select one.**

□ Never (0% of systematic reviews)

□ Rarely (25% of systematic reviews)

□ Sometimes (50% of systematic reviews)

□ Mostly always (75% of systematic reviews)

□ Always (100% of systematic reviews)

**7. Which of the following methods are used to evaluate an assessment of selective reporting bias to ensure completeness? Select all that apply.**

□ Not applicable (assessments not reviewed)

□ Editors confirm that the Cochrane risk of bias tool has been completed for each included RCT

□ Editors review completed Cochrane risk of bias tool for compatibility between the review authors' judgment of risk of selective reporting bias and the support for the judgment (i.e., low risk when stated that all specified outcomes reported)

□ Editors select a sample of trials to which the Cochrane risk of bias tool has been applied to independently

verify the accuracy of the assessment of risk of selective reporting bias

□ Other. Please elaborate: ____________________

**8. Does your editorial team require review authors to contact trial authors regarding the completeness of published data? Select one.**

□ Yes

□ No

□ Sometimes. Please elaborate: ____________________

**9. Does your editorial team require review authors to provide a record of this communication (or communication attempts) for review by editors? Select one.**

□ Yes

□ No

□ Sometimes. Please elaborate: ____________________

**10. The Cochrane Handbook recommends review authors to consider key outcomes that are routinely measured in trials regarding their topic of interest, and to construct a matrix or chart recording which trials considered for the review measure these outcomes and which do not.(1) Does your editorial team require review authors to construct such a matrix of study outcomes to better assess for unreported outcomes in ALL trials considered for inclusion in the systematic review? Select one.**

□ Yes

□ No

□ Sometimes. Please elaborate: ____________________

(1) Higgins JPT, Altman DG, Sterne JAC (editors). Chapter 8: Assessing risk of bias in included studies. In: Higgins JPT, Green S (editors). Cochrane Handbook for Systematic Reviews of Interventions Version 5.1.0 (updated March 2011). The Cochrane Collaboration, 2011. Available from www.cochrane---handbook.org

**11. When considering a trial for inclusion in a systematic review, does your editorial team require review authors to search for the corresponding trial protocol to assess for discrepancies in outcomes between the protocol and final published trial? Select one.**

□ Yes

□ No

□ Sometimes. Please elaborate: ____________________

**12. Does your editorial team independently verify that differences from trial protocols to published trials are accurately reported in the risk of bias assessment? Select one.**

□ Yes

□ No

□ Sometimes. Please elaborate: ____________________

**13. Does your editorial team require review authors to incorporate the assessment of selective reporting bias into the Results and/or Discussion sections of the systematic review (i.e., comment on its magnitude and direction)? Select one.**

□ Yes

□ No

□ Sometimes. Please elaborate: ____________________

**14. Estimate the percentage of systematic reviews initially submitted for peer review for which review authors are asked to redo or revise an assessment of selective reporting bias. Provide a numerical value from 1 to 100 (%).**

**15. For what reasons are review authors asked to redo an assessment of selective reporting bias? Rank responses according to frequency of reason for revision without duplicating numbers (1 = most frequent reason, 2 = second most frequent reason, etc). Select N/A if response is inapplicable. If review authors are never asked to redo assessments, please rank "None of the above, never asked to redo assessments" as 1 and all other responses as N/A.**

□ Cochrane risk of bias tool not completed for each included RCT

□ Verification of assessment within Cochrane risk of bias tool reveals inconsistencies across trials (example: criteria for judgment of "low risk" inconsistent from trial to trial)

□ Verification of assessment within Cochrane risk of bias tool reveals discrepancies in judgment of risk between review author and editor (example: review author listed "low risk" what editor would deem "high risk")

□ Review author's interpretation of the impact of selective reporting bias is inadequate or absent

□ No search for trial protocols performed

□ No trial authors contacted

□ No matrix of trials and measured outcomes created

□ None of the above, never asked to redo assessments

### Assessment of Risk of SRB on Level of the Systematic Review

**16. Does your editorial team assess the potential for review authors selectively including trials in analyses (i.e., is consideration given to whether the subset of data included in the systematic review fully represents available trial data)? Select one.**

□ Yes

□ No

□ Sometimes. Please elaborate: ____________________

**17. Does your editorial team look for discrepancies in the outcomes originally defined for analysis in the systematic review protocol and those analyzed in the final systematic review? Select one.**

□ Yes

□ No

□ Sometimes. Please elaborate: ____________________

**18. If discrepancies in outcomes defined in the protocol versus the final systematic review are noted, what is the minimum amount of information your editorial team requires review authors to provide in the systematic review to address these changes? Select all that apply.**

□ Not required to comment on changes in outcomes

□ Ensured that the changes in outcomes are reported in the Methods section

□ Ensured that a justification for each change is included within the Methods section

□ Insisted that all original analyses be reported in the final systematic review

□ Other. Please elaborate: ____________________

### Assessment of Risk of SRB in Updates of Systematic Review

**19. How is the assessment of selective reporting bias approached when updates of systematic reviews are submitted for peer review? Select all that apply.**

□ No emphasis placed on assessment of risk of bias

□ Cochrane risk of bias tool must be applied only to new RCTs being introduced into the systematic review

□ Cochrane risk of bias tool must be applied to all RCTs

□ Updated risk of bias assessment must be incorporated into the Results and/or

□ Discussion sections in terms of the likely direction and magnitude of bias

□ Other. Please elaborate: ________________

### Importance to Review Authors

**20. We would like to know how well you feel your group's review authors understand selective reporting bias and how well-equipped they are to address it in systematic reviews. For each of the factors listed in the left column, select the numbered descriptor that gives the most fitting description, in your opinion, of review authors submitting to your review group.**

| | **Not at all** | **To a little extent** | **To some extent** | **To a moderate extent** | **To a large extent** |
|---|---|---|---|---|---|
| Review authors' degree of understanding of the *existence* of selective reporting bias | | | | | |
| Review authors' degree of understanding of the *implications* of selective reporting bias | | | | | |
| Review authors' degree of *competency in completing assessments* of selective reporting bias | | | | | |
| Review authors' degree of *motivation to complete assessments* of selective reporting bias | | | | | |

**General**

**21. How often has your review group sought guidance from the Cochrane Bias Methods Group (CBMG) regarding the assessment of selective reporting bias in the past year?**

- ◻ Unknown
- ◻ Never
- ◻ One or two times
- ◻ Frequently. Quantify: ________________
